# Altered Expression of Calpastatin by Hypoxia Regulates Trophoblast Cell Function through Mitochondria Associated Endoplasmic Reticulum Membranes

**DOI:** 10.1007/s43032-025-01995-4

**Published:** 2025-10-29

**Authors:** Cui Zhang, Jingjing Jiang, Hongfang Kong, Xuyuan Ma, Haiyan Li, Hong Xin

**Affiliations:** 1 Department of Obstetrics, Hebei General Hospital, Hebei Medical University, Shijiazhuang, China; 2 Department of Obstetrics, The Second Hospital of Hebei Medical University, Hebei Medical University, Shijiazhuang, China; 3 Department of Obstetrics and Gynecology Ultrasound, The Second Hospital of Hebei Medical University, Hebei Medical University, Shijiazhuang, China

**Keywords:** Preeclampsia, Mitochondria, Endoplasmic reticulum, Oxidative stress, Trophoblasts

## Abstract

Preeclampsia (PE), a severe pregnancy complication, arises from placental hypoxia-induced mitochondrial and endoplasmic reticulum (ER) oxidative stress, contributing to inadequate spiral artery remodeling and endothelial dysfunction. Calpastatin, a mitochondrial protective protein, mitigates oxidative stress-related pathologies, but its role in PE remains unclear. This study investigated the effects of Calpastatin on trophoblast cellular proliferation, migration, invasion, apoptosis, and the expression of autophagy protein (PINK1), mitochondrial dynamics protein (Mfn2), ER stress protein (GRP78), ATP, Ca^2+^, and mitochondrial membrane potential under hypoxia using transfected HTR8-SVneo cells. Calpastatin overexpression significantly enhanced proliferation, migration, and invasion while reducing apoptosis (*P* < 0.05); knockdown inversely affected these parameters under normoxic conditions. Under hypoxia, overexpression further amplified proliferation and migration (*P* < 0.01), whereas knockdown reduced migration at 48 h (*P* = 0.04) but not proliferation. Invasion decreased and apoptosis increased in both groups (*P* < 0.05). Calpastatin overexpression upregulated PINK1, downregulated Mfn2/GRP78, increased ATP and mitochondrial membrane potential, and reduced Ca^2+^. Conversely, knockdown suppressed Pink1/Parkin, elevated Mfn2/Drp1/GRP78, decreased ATP, and increased Ca^2+^ and mitochondrial depolarization (*P* < 0.05). These findings demonstrate calpastatin promotes trophoblast function by maintaining mitochondrial-ER contact sites stability and ATP production, Ca^2+^ homeostasis, and mitophagy mechanism, suggesting its critical role in PE pathogenesis.

## Introduction

Preeclampsia (PE) is a leading cause of maternal and fetal morbidity and mortality, characterized by hypertension and multi-organ dysfunction after 20 weeks of gestation.The pathophysiology of PE involves placental dysfunction resulting from abnormal vascular remodeling and a systemic inflammatory response [1]. Persistent placental hypoxia in early pregnancy by causing mitochondrial dysfunction and endoplasmic reticulum stress (ERS). This compromises trophoblast invasion and subsequent remodeling of the uterine spiral arteries, resulting in reduced placental perfusion, ischemia and hypoxia. The ensuing placental dysfunction and trophoblast abnormalities drive the development of systemic PE manifestations [2, 3].

Ischemia-hypoxia leads to calpastatin imbalance, causing mitochondrial dysfunction and oxidative stress [4],which are common features in PE placentas [5–8]. Studies by Goto S and Takano J et al. [9] demonstrated that calpain, a substrate of calpastatin, plays a critical role in fetal growth restriction (FGR) and maintaining placental integrity for embryonic survival [10].Current evidence lacks mechanistic links between calpastatin and trophoblast cells function. We hypothesize that calpastatin preserves trophoblast function by stabilizing mitochondrial integrity and attenuating mitochondrial-ER stress cascades under PE-mimicking hypoxia.This study addresses this critical gap by examining the impact of calpastatin overexpression and knockdown on abnormalities in trophoblast biological behaviors and mitochondrial homeostasis in a hypoxia-induced PE cell model.

## Materials and Methods

### Immunofluorescence Staining

Prior to cell experiments, we determined the localization of calpastatin in placental trophoblast cells from PE patients. We selected 30 cases each of PE and normal placental tissues. This sample size was calculated using G*Power 3.1 software based on a pilot experiment. PE diagnosis complied with the standards of the American College of Obstetricians and Gynecologists [11]. Control subjects were matched for gestational age and excluded individuals with chronic hypertension, immune system disorders, diabetes, or other diseases pathologically associated with PE.The study was approved by the hospital’s Ethics Committee (Approval No.: 2024-LW-133). Placental sections were deparaffinized in xylene substitutes (Eco-friendly Dewaxing Solution I/II/III), rehydrated through an ethanol series, and subjected to heat-mediated antigen retrieval in EDTA buffer (pH 8.0) using microwave irradiation. After blocking with 3% BSA, sections were incubated overnight at 4 °C with rabbit anti-calpastatin primary antibody (Abcam; 1:1000). Following PBS washes, sections were incubated with Alexa Fluor 488-conjugated goat anti-rabbit secondary antibody (Servicebio; 1:400) for 50 min at room temperature in the dark. Nuclei were counterstained with DAPI. Slides were mounted with antifade medium and imaged using a Nikon Eclipse C1 fluorescence microscope.

### Cell Line Selection and Culture

The trophoblast cell line HTR8/SVneo was purchased and cultured in complete medium prepared by mixing (RPMI-1640 medium, fetal bovine serum,penicillin–streptomycin; Gibco, USA; 100:10:1 ratio). Cells were incubated in a 37 °C, in 5% CO_2_ incubator. For hypoxic treatment, cells were placed in an incubator containing 95% N_2_, 5% CO_2_, and 1% O_2_, and hypoxic/reoxygenation (H/R) invoved reoxygenation at 21% O_2_ partial pressure.

### Generation of Stable Transfected Cell Lines

pc-DNA, pc-DNA-CAST, pLKO.1-puro, sh-RNA1, sh-RNA2, and sh-RNA3 were purchased from Guangzhou FuNeng Gene. Sh-RNA1, sh-RNA2, and sh-RNA3 represent three different sequences targeting calpastatin; sh-RNA1 exhibiting the highest inhibition efficiency, was selected for subsequent experiments. According to the manufacturer's instructions, cells in logarithmic growth were seeded in 6-well plates. At 80–90% confluence, transfection was performed using Lipofectamine 3000 transfection reagent (Invitrogen, USA). After 6 h, the medium was changed. Functional analyses and measurements were performed 24–48 h post-transfection. All experiments were conducted with 3 biological replicates,each with 3 technical replicates.

### Western Blotting

Cells were lysed with high-efficiency RIPA buffer containing PMSF (Solarbio, R0010). Total protein concentration was measured using the BCA assay (Abbkine, KTD3001). Protein samples were diluted to equal concentration, mixed with 5 × loading buffer, boiled at 97 °C for 4 min, and stored at −80 °C. For each group, 80 μg of total protein was separated by SDS-PAGE and transferred to a PVDF membrane. The membrane was blocked with 5% skim milk and incubated overnight at 4 °C with primary antibodies against calpastatin (1:1000, Abcam, ab28252), HIF-1α (1:800, Zenbio, 340462), VEGF-A (1:800, Zenbio, R26073), PINK1 (1:800, Wanleibio, WL04963), Mfn2 (1:1000, Wanleibio, WL06394), GRP78 (1:1000, Wanleibio, WL03157), and GAPDH (1:2000, Servicebio, P16858). The next day, the membrane was incubated for 1 h with DyLight800 goat anti-rabbit IgG (1:5000, Proteintech). Protein bands were visualized using an infrared fluorescence imaging system (Gene Company Limited, Odyssey CLx), Band intensity was quantified using Image J software (NIH, USA), with GAPDH as the loading control.

### Trophoblast Cell Function Assays

#### Cell Proliferation Assay

Proliferation was assessed using the CCK-8 kit (Abbkine, BMU106-CN).Cells were seeded in 96-well plates and subjected to 12 h of hypoxia before transfection. At 0, 24, and 48 h post-transfection, 10 μL of CCK-8 solution was added to each well, After 2 h incubation, absorbance at OD450 nm was measured.

#### Cell Migration Assay

Cells were seeded in 6-well plates. A scratch was made using a 200 μL pipette tip along a marked. After 12 h of hypoxia, the cells were transfected. Images were taken at 0, 24, and 48 h post-transfection using an inverted microscope (ZEISS AXIO). The wound area was calculated using Image J software.

#### Cell Invasion Assay

60 μL of diluted Matrigel was placed in the Transwell chamber and incubated for 3 h. After transfection, 200 μL of cell suspension (2.5 × 10^5^ cells/mL) was added to the upper chamber. The lower chamber contained 500μL medium containing 10% FBS. Cells were incubated for 24–48 h. Non-invasive cells on the upper surface were removed with a cotton swab. Cells invading the lower surface were fixed with 4% paraformaldehyde for 30 min, stained with 0.1% crystal violet for 15 min, washed twice with PBS, and counted under a microscope.

#### Cell Apoptosis Assay

Apoptosis was assessed using the Annexin V-FITC/7AAD Apoptosis Kit (Elabscience, E-CK-A212). After 48 h of transfection, cells were resuspended in 500 μL 1X Annexin V Binding Buffer. 5 μL of Annexin V-FITC (5μL) and 7-AAD (5μL, 100 μg/mL) were added and mixed. Cells were incubated in the dark for 20 min. Apoptosis was analyzed by flow cytometer (Flow Jo 10.8.1 software). Viable cells (Annexin V^-^/7-AAD^-^), early apoptotic cells (Annexin V^+^/7-AAD^-^), late apoptotic/necrotic cells (Annexin V^+^/7-AAD^+^), and dead cells (Annexin V^-^/7-AAD^+^) were quantified.

### Mitochondrial and Endoplasmic Reticulum Related Indicators

#### ATP Content Measurement

ATP content was measured using the ATP Assay Kit (Mlbio, ATP-W48-N1620) based on the phosphomolybdate colorimetric method. After 48 h transfection, cells were collected.Half the sample was used for BCA protein measurement. Cells were counted, treated with acidic extraction solution and lysed by ultrasonication on ice. An equal volume of alkaline extraction solution was added. Samples were centrifuged (8,000 rpm,10 min, 4 °C). Supernatant was incubated with color reagent (37 °C, 20 min). Absorbance at 700 nm was measured, and ATP content calculated using a standard formula.

#### Calcium Ion Content Measurement

Ca^2+^ content was measured using the Ca^2+^ Assay Kit (Nanjing Jiancheng, C004-2–1 MTB) based on the MTB microplate method. After 48 h transfection, cells were collected.Half of the sample was used for BCA protein measurement. Cells were washed 1–2 times with saline (0.5-1 mL), centrifuged (1,000–2,000 rpm), disrupted by ultrasonication on ice bath and centrifuged (2,000 rpm,10 min). Supernatant was mixed with MTB solution, alkaline solution, and protein clearing solution.Absorbance at 610 nm was measured, and Ca^2+^ content was calculated.

#### Mitochondrial Membrane Potential Measurement (JC-1 Staining)

Mitochondrial membrane potential(MMP) was assessed using the JC-1 Mitochondrial Membrane Potential Detection Kit (UElandy, J6004S, J6004L). After 48 h of transfection, 1 mL of complete culture medium was added to the cells, followed by JC-1 staining working solution. The cells were incubated for 20 min, washed with 1 mL of pre-cooled 1 × Assay Buffer, and observed under a fluorescence microscope.

### Statistical Analysis

Statistical analysis was performed using SPSS 24.0 software (SPSS, Chicago, Illinois). Data from repeated experiments and technical replicates are presented as mean ± standard deviation (SD) or median (interquartile range). Nonparametric tests or Student’s t tests should be used for intergroup comparisons. Data processing and figure generation were performed using GraphPad Prism 7. A P-value of less than 0.05 was considered statistically significant.

## Results

### Localization of Calpastatin in the PE

**Fig. 1:**
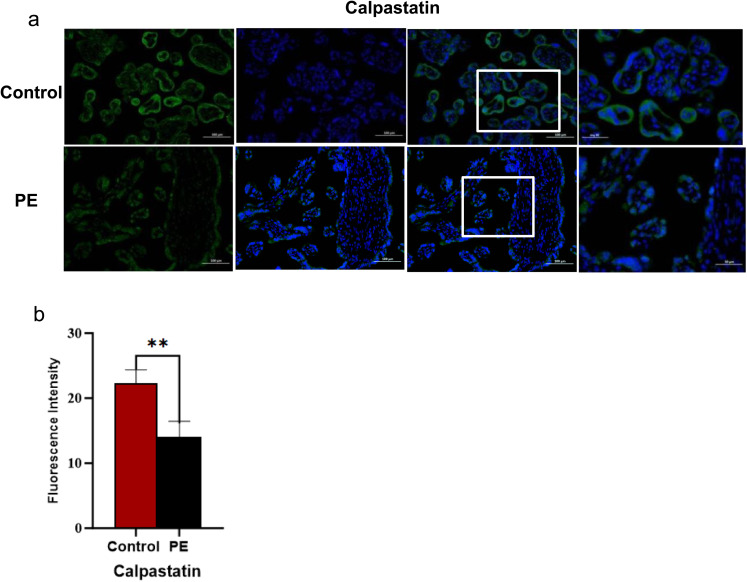
**a** showed immunofluorescence staining of calpastatin. micrographs of human placenta trophoblast cell stained in controls and PE. The nuclei were stained with DAPI (blue). SY3 (Green color) were stained and represents the localization of calpastatin. Scale bar 100 μm, 50 μm. **b** was quantitative analysis bar charts of the fluorescence intensities of calpastatin, showing the differences among the control and PE groups. ** represents *P* < 0.01

Calpastatin expression was localized to syncytiotrophoblast cells in placental tissue (Fig. 1a). Fluorescence intensity was significantly lower in PE group compared to controls (*P *< 0.001) (Fig. 1b).PE newborns had lower birth weights than controls(*P* = 0.04; Table 1).No statistically differences were observed in maternal age, prepregnancy weight, parity, gestational age at delivery, and placental weight (*P* > 0.05).

**Table 1:** Baseline characteristics

Baseline	Control (*n* = 30)	PE (*n* = 30)	*P*
Age (years)	31.60 ± 5.05	30.81 ± 4.08	0.43
Pre-pregnancy BMI (kg/m^2^)	24.63 ± 4.4	27.26 ± 5.29	0.07
Primiparous (n,%)	2(1.5)	3(3)	0.42
Gestational age at delivery (weeks)	31.47 ± 2.0	32.29 ± 1.57	0.05
Birth weight (g)	1766 ± 533.99	1637 ± 391.07	0.04*
Placenta weight (g)	400(50)	430(170)	0.63

### Expression of HIF-1α and VEGF-A Under Hypoxia and Reoxygenation

**Fig. 2:**
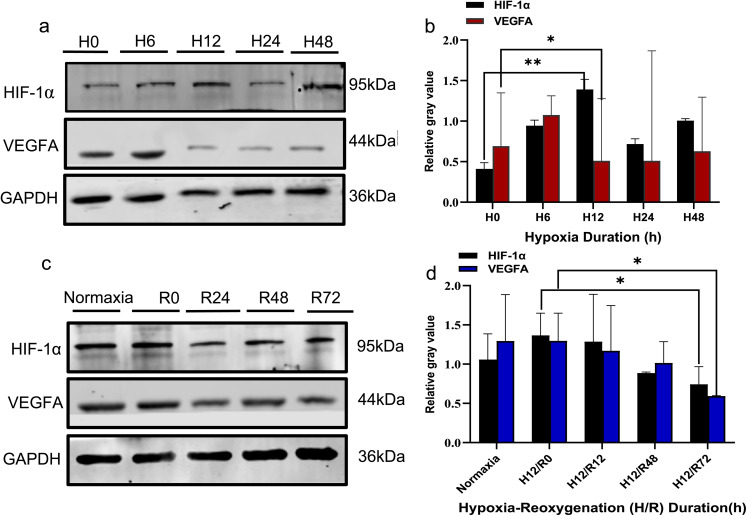
**a** and **c** represents Western blotting of HIF-1α and VEGFA protein expression under hypoxia over time (0 h, 6 h, 12 h 24 h, 48 h) and different reoxygenation time(0 h, 24 h, 48 h, 72 h) after 12 h of hypoxia. From top to bottom are protein bands of HIF-1α (95 kDa), VEGFA (44 kDa), and internal reference GAPDH (36 kDa). **b** and **d** represents the relative gray-scale values of the expression levels. And gray bar represents VEGF-A, black bar represents HIF-1α. Data represent mean ± SD of > 3 independent experiments with ≥ 3 technical replicates each. ** indicates extremely significant difference (*P* < 0.01). * indicates significant difference (*P* < 0.05)

We investigated HIF-1α and VEGF-A proteins levels under varying durations of hypoxia (6 h,12 h) and reoxygenation (24 h,48 h,72 h after 12 hypoxia). HIF-1αlevels peaked at 12 h hypoxia and subsequently declined. VEGF-A levels were elevated at 6 h hypoxia and gradually decreased with extended hypoxia (*P* < 0.01). Following 12 h hypoxia, reoxygenation gradually restored HIF-1α and VEGF-A levels towards normal. At 72 h reoxygenation, levels remained lower than at 12 h hypoxia without reoxygenation (*P* < 0.05; Fig. 2a-d).Consequently, 12 h hypoxia was selected as the experimental time point.

### Validation of Cell Transfection Efficiency

**Fig. 3:**
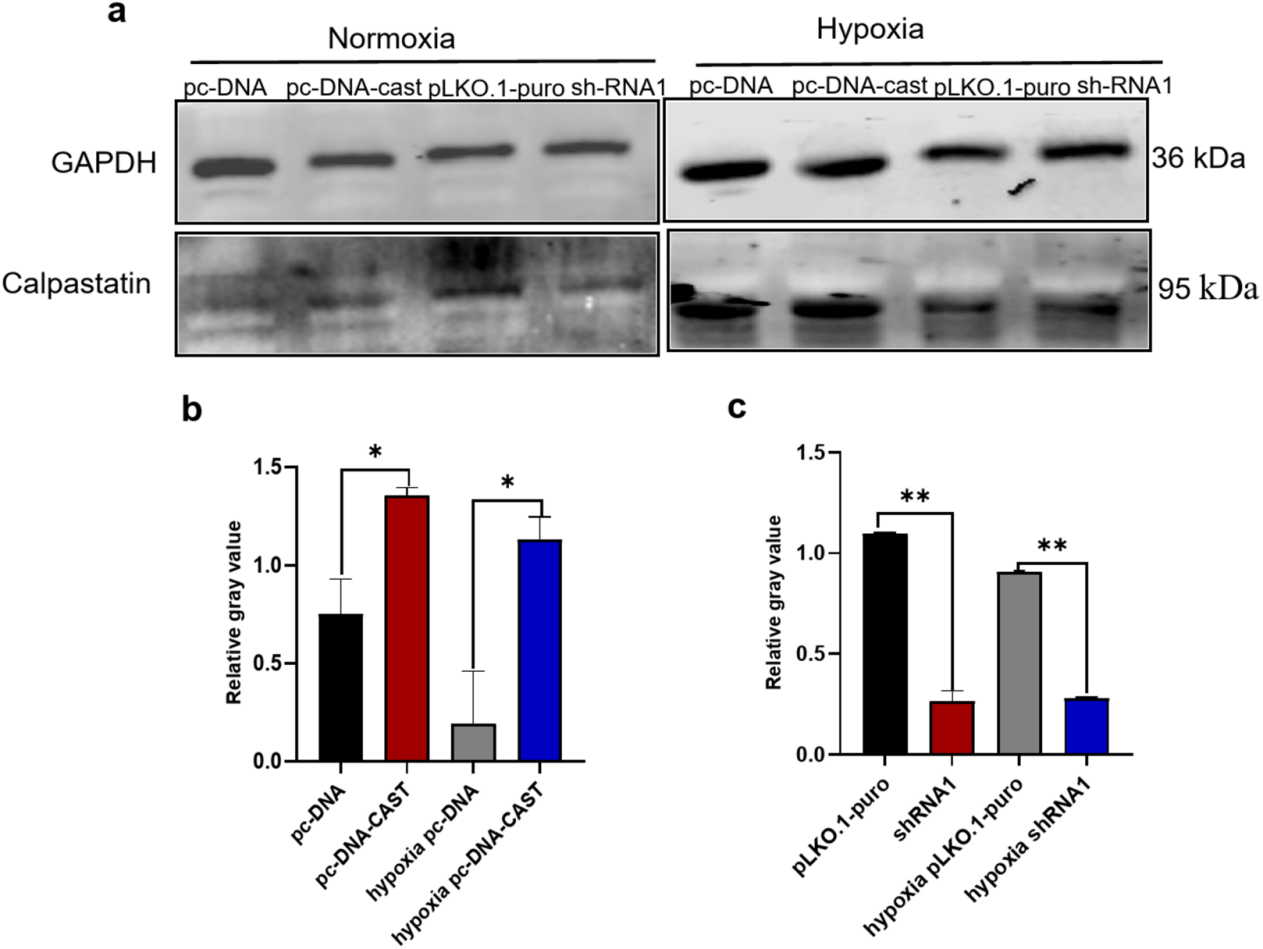
**a** shows the Western blotting of Calpastatin protein expression under normoxic and hypoxic conditions with different transfection treatments. GAPDH (36 kDa) serves as the internal reference, The transfection treatments include pc-DNA, pc-DNA-CAST, pLKO.1-puro, and shRNA1. Data represent mean ± SD of > 3 Transfections replicates with ≥ 3 technical replicates. **b** and **c** illustrate the relative expression levels of Calpastatin protein. **P < 0.01; *P < 0.05

Calpastatin protein expression was assessed 48 h post-transfection. Under normoxia, expression was significantly higher in the pc-DNA-CAST group than the pc-DNA control (P = 0.004). Similarly, under hypoxia, expression was higher in the hypoxia pc-DNA-CAST group versus control (P = 0.005). Calpastatin levels in both normoxic and hypoxic sh-RNA1 groups were significantly lower than their respective controls (*P* < 0.001 and *P* = 0.005, respectively; Fig. 3A-C).

### Impact of Calpastatin Knockdown on Trophoblast Cell Function under Hypoxia

#### Effect on Cell Proliferation by CCK-8 Assay

**Fig. 4:**
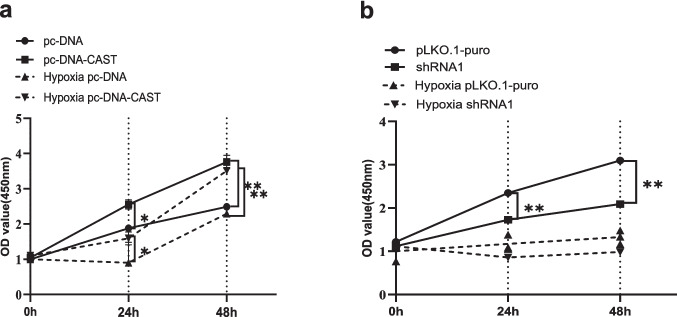
OD values (450 nm) representing the cell proliferation over time (0 h, 24 h, 48 h) under different transfection treatments and normoxic, H/R conditions by CCK-8 assay. Triplicate wells were measured for absorbance at each test point. **P < 0.01; *P < 0.05

Calpastatin overexpression significantly enhanced trophoblast cell proliferation at 24 h under both normoxia (*P* = 0.046) and hypoxia (*P* = 0.02). Proliferation further increased at 48 h (*P* = 0.007 and *P* = 0.0011, respectively). Conversely, Calpastatin knockdown significantly reduced proliferation at 24 h and 48 h under normoxia (*P* = 0.0039 and *P* < 0.0001, respectively). However, under hypoxia, knockdown did not significantly affect proliferation at either time point (*P* = 0.44 and *P* = 0.32; Fig. 4a, b).

#### Effect on Cell Migration by Wound Healing Assay

**Fig. 5:**
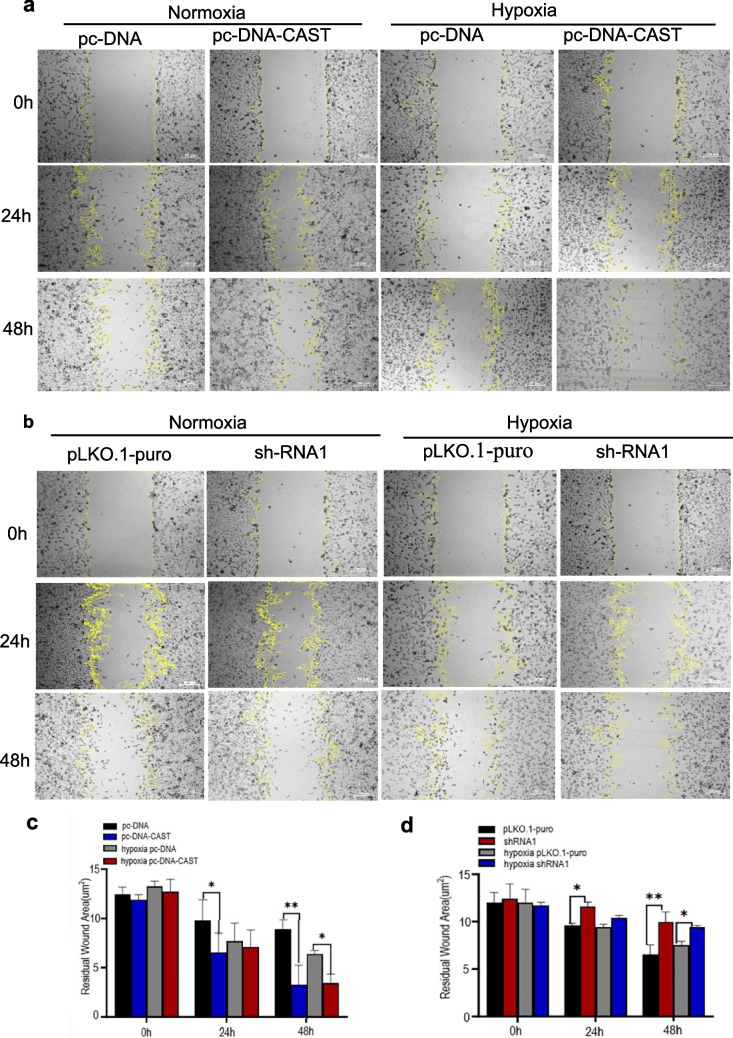
**a** and **b** represent images of Wound healing assay for different transfection groups(pc-DNA, pc-DNA-CAST, pLKO.1-puro, and sh-RNA1) at 0 h, 24 h, and 48 h under normoxic and H/R conditions to evaluate cell migration function. The yellow lines mark the edges of the scratches (*n* = 3 technical replicates). **c** and **d** represent quantification results of scratch areas (μm^2^) shown in **a** and **b**. Wound healing assays were performed in triplicate (*n* = 3 biological replicates) with 3 technical replicate wells per condition. Quantification data represent mean ± SD. **P < 0.01; *P < 0.05

Calpastatin overexpression significantly enhanced migration at 24 h and 48 h under normoxia (*P* = 0.02 and *P* = 0.0002, respectively). Under hypoxia, migration was significantly higher only at 48 h (*P* = 0.04). Knockdown significantly impaired migration at 24 h and 48 h under normoxia (*P* = 0.01 and *P* = 0.0015, respectively) and at 48 h under hypoxia (*P* = 0.04; Fig. 5a-d).

#### Effect on Cell Invasion by Transwell Assay

**Fig. 6:**
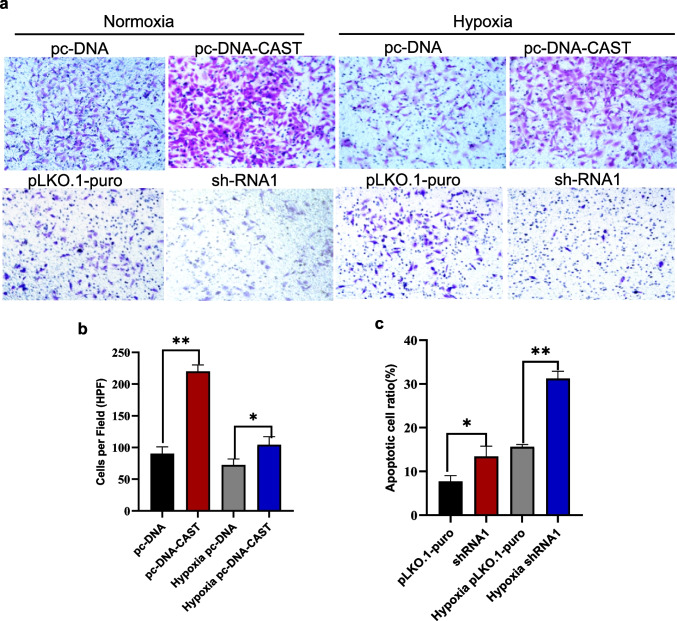
**a** Staining images of cell invasion assays for different transfection groups (pc-DNA, pc-DNA-CAST, pLKO.1-puro, sh-RNA1) under normoxic and hypoxic conditions to observe cell invasion. by Transwell assay. **b** and **c** represent the number of invasive cells in the four transfection groups under normoxic and H/R conditions. The abscissa represents different transfection groups, and the ordinate represents the number of invasive cells. Cell invasion was performed in triplicate (*n* = 3 biological replicates) with 3 technical replicate wells per condition. Quantification data represent mean ± SD. **P < 0.01; *P < 0.05

Calpastatin overexpression significantly enhanced cell invasion under both normoxia (*P* < 0.001) and hypoxia (*P* = 0.025). Knockdown significantly reduced invasion capacity under both conditions (*P* = 0.006 and *P* < 0.001, respectively) (Fig. 6a-c).

#### Effect of Calpastatin on Cell Apoptosis by Flow Cytometry

**Fig. 7:**
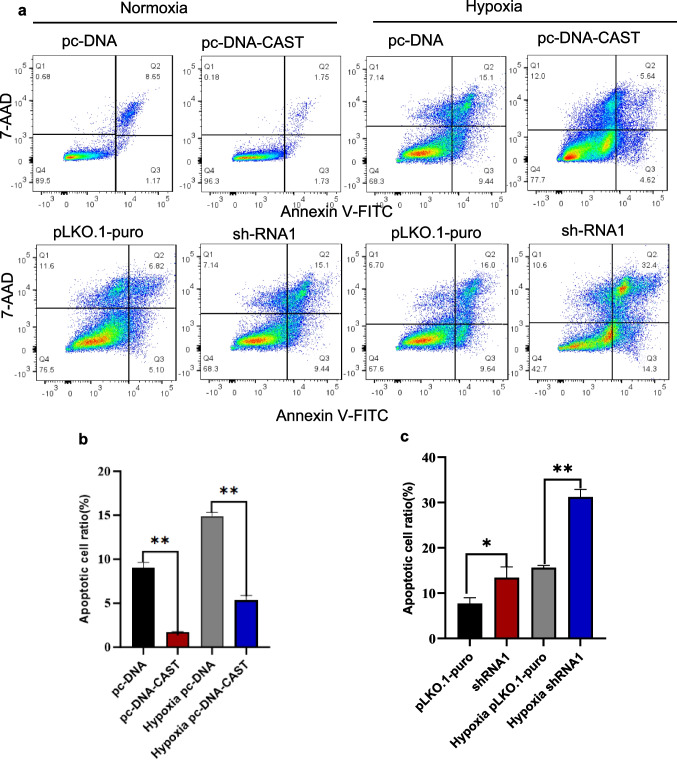
**a** Flow cytometry scatter plots of cell apoptosis detected by Annexin V-FITC and 7-AAD double-staining. The plots show the cell apoptosis status under different transfection conditions and normoxic, and H/R. The lower right quadrant Q3 represents early apoptotic cells; the upper right quadrant Q2 indicates late apoptotic or necrotic cells. The upper left quadrant Q1 corresponds to bare nuclear cells while the lower left quadrant Q3 represents viable cells. Quantification of apoptotic cells (mean ± SD, *n* = 3 biological replicates with 20,000 events acquired per replicate. **b** and **c** represents the apoptotic cell ratios of across treatment groups. **P < 0.01; *P < 0.05

Calpastatin overexpression significantly reduced apoptosis under both normoxia and hypoxia (*P* < 0.001 for both). Knockdown significantly increased apoptosis (*P* = 0.002 and *P* < 0.001, respectively) (Fig. 7a-c).

### Impact of Calpastatin on Mitochondrial and Endoplasmic Reticulum Function under Hypoxia

#### Effect on Mitochondrial and ER-Related Protein Expression

**Fig. 8:**
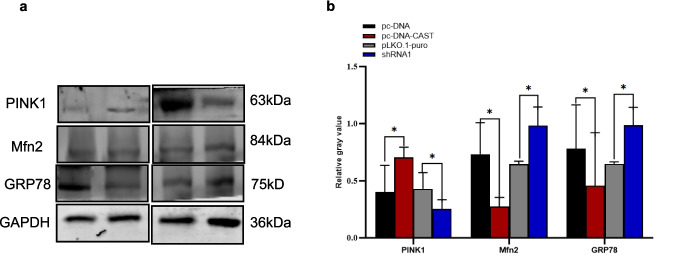
Effect of MAMs-associated protein relative expression level in hypoxia/reoxygenation by western bloting **a** Western blotting analysis. This experiment detected the expression levels of relevant proteins in cells under different transfection conditions. The transfection conditions included pc-DNA, pc-DNA-CAST, pLKO.1-puro, and shRNA1. The proteins detected were PINK1 (63 kDa),, Mfn2 (84 kDa), GRP78 (75 kDa). GAPDH (36 kDa) was used as an internal reference protein to normalize the protein loading amount. Transfections were performed in > 3 biological replicates (independent experiments) using consistent DNA/lipid ratios, with ≥ 3 technical replicates (parallel wells) per condition. **b** Quantification data (mean ± SD) were normalized to GAPDH loading control. *P < 0.05

Under hypoxia, Calpastatin overexpression increased the autophagy-related protein PINK1 while reducing the mitochondrial fusion protein Mfn2 and the ER stress marker GRP78 (*P* < 0.05). Conversely, knockdown decreased PINK1 expression, increased Mfn2 levels, and elevated GRP78 expression (*P* < 0.05) (Fig. 8a, b).

#### Effect on Cellular ATP Content

**Fig. 9:**
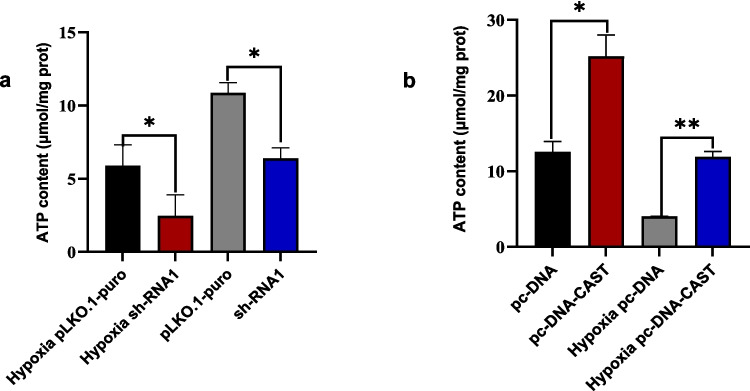
**a** and **b** showing the ATP content (in μmol/mg prot) under different conditions. The conditions include pc-DNA, pc-DNA-CAST, hypoxia pc-DNA, hypoxia pc-DNA-CAST, pLKO.1-puro, shRNA1, hypoxia pLKO.1-puro, and hypoxia shRNA1 (*n* = 3 technical replicates, mean ± SD). *P < 0.05, **P < 0.01

Calpastatin overexpression significantly increased ATP content under both normoxia (*P* = 0.03) and hypoxia (*P* = 0.004). Knockdown reduced ATP levels under both conditions (*P* = 0.013 and *P* = 0.023, respectively) (Fig. 9a, b).

#### Effect on Cellular Ca^2+^ Content

**Fig. 10:**
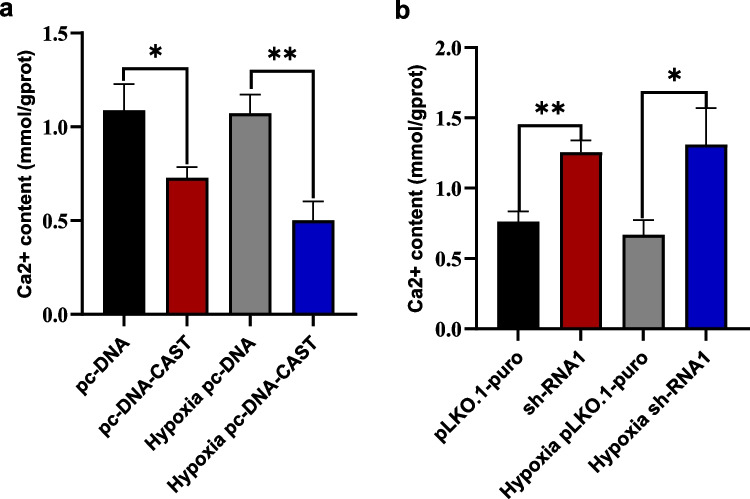
**a** and **b** showing the Ca2 + content (in mmol/g prot) by MTB microplate method under different conditions. The conditions include pc-DNA, pc-DNA-CAST, hypoxia pc-DNA, hypoxia pc- DNA-CAST, pLKO.1-puro, shRNA1, hypoxia pLKO.1-puro, and hypoxia shRNA1 (*n* = 3 technical replicates, mean ± SD).**P < 0.01; *P < 0.05

Calpastatin overexpression significantly reduced calcium ion content under normoxia (*P* = 0.023) and hypoxia (*P* < 0.01). Knockdown increased Ca^2+^ levels under both conditions (*P* < 0.01 and *P* = 0.02, respectively) (Fig. 10a, b).

#### The Effect of Calpastatin Knockdown on Trophoblast Cell Mitochondrial Membrane Potential (MMP)

**Fig. 11:**
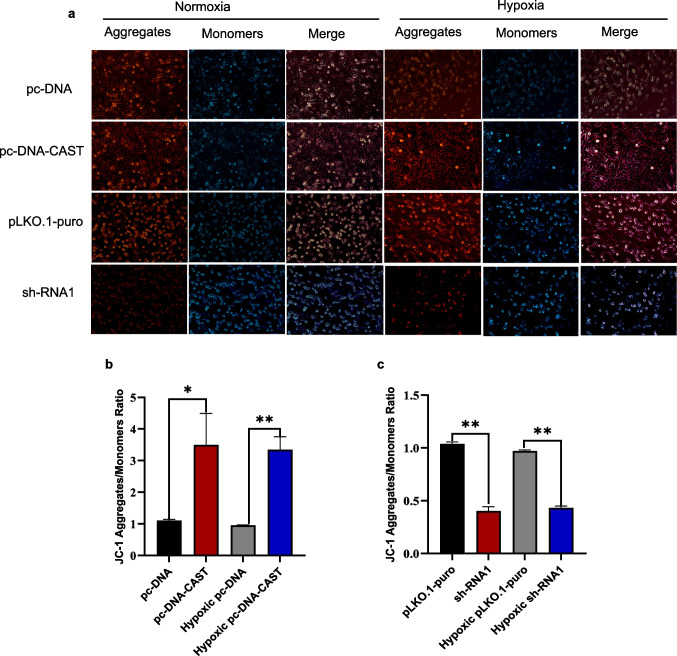
Effect of mitochondrial membrane potential(MMP) in hypoxia/reoxygenation by JC-1. **a** Fluorescence microscopy images of cells under normoxia and hypoxia. Shows Aggregates (red), Monomers (green), and Merged views for pc- DNA, pc-DNA - CAST, pLKO.1-puro, and sh-RNA1 groups. **b** and **c** Bar graph of red-to-blue fluorescence ratio. Data represent mean fluorescence intensity ratio (red/blue) ± SD from three technical replicates experiments. *P < 0.05, ** *P* < 0.01

To evaluate the impact of calpastatin on MMP, JC-1 staining was employed. In normal cells, the MMP is relatively high. JC-1 aggregates in the mitochondrial matrix to form polymers, emitting red fluorescence. In apoptotic cells, the MMP decreases, and JC-1 exists in a monomeric form, emitting green fluorescence. Calpastatin overexpression resulted in enhanced fluorescent aggregate signals and weakened monomeric signals, whereas knockdown led to reduced aggregates accompanied by increased monomers, indicating that overexpression elevated MMP, while knockdown caused its decline. Quantitative analysis confirmed a significantly higher aggregate/monomer ratio in the overexpression group compared to controls (*P* = 0.013; *P* = 0.0005), while the knockdown group exhibited a markedly lower ratio (*P* < 0.05; Fig. 11a, b).

## Discussion

PE is linked to placental syncytiotrophoblast stress and hypoxia, resulting from dysfunctional uterine tolerisation to allogeneic trophoblast, impaired placentation, inadequate remodeling causing persistent placental ER oxidative stress, and maternal factors likes chronic pre-pregnancy diseases [12]. Chronic pre-pregnancy diseases, such as diabetes, severe anemia, Systemic Lupus Erythematosus(SLE), hypertension predispose to PE by affecting early placental implantation, leading to early hypoxia. Molecular mechanisms of hypoxic differentiation are essential for trophoblast function and placentation. Early pregnancy hypoxia differentiation,proliferation, invasion, and differentiation; sustained low oxygen (O_2_) levels, can lead to complications such as PE [3]. Fuenzalida et al. demonstrated that hypoxia or H/R models reflect PE pathological processes, making them suitable in vitro models [13].

During the first trimester, low oxygen partial pressures (1–3% pO_2_) are vital for placental development and regulates trophoblast differentiation [14, 15]. We utilized a H(1% pO_2_)/R (21%pO_2_) to model PE clearly revealing the biological functions of trophoblast cells in our team [16] and 1–3%pO_2_ also serves as a model for preeclampsia/FGR or trophoblast differentiation and migration in vitro [17]. Clinically, placental stress occurs in hypoxia, and syncytiotrophoblast cells produce soluble fms-like tyrosine kinase-1 (sFlt-1), which inhibits VEGF-A and placental growth factor (PLGF) and ultimately inducing endothelial dysfunction in PE [12]. In this study we will select the time of H/R according to HIF-1α and VEGF-A levels. VEGF-A expression was decreased and HIF-1α increased with prolonged hypoxia. So we selected 12 h hypoxia as the critical time point. Under 12 h hypoxia, after 72 h reoxygenation, HIF-1α and VEGF-A levels were lower than those in hypoxic cells without reoxygenation, indicating that reoxygenation still affected the cells.

Calpastatin indirectly participates in the regulation of intracellular Ca^2+^ homeostasis by inhibiting the activity of calpain, which is crucial for promoting blood flow restoration by inhibiting inflammatory and promoting vascular regeneration [18]. While its protective role in calpastatin in cardiovascular and neurological disease is established [19, 20].emerging evidence links abnormal calpastatin-mediated Ca^2+^ regulation to PE [21]. We found calpastatin was predominantly expressed in syncytiotrophoblast cells, which differentiate into extravillous trophoblast (EVT) migrating to spiral arteries to replace vascular endothelial cells, completing vascular remodeling [22]. This suggests calpastatin influence trophoblast cell function, leading to vascular remodeling. Its low expression in PE and association with low neonatal birth weight further support this role. Lu et al. found increased mitochondrial fission caused by abnormal Ca^2+^ efflux leads to vascular smooth muscle cells (VSMCs) transformation and the impaired spiral artery remodeling [23].The most common calcium signaling pathway involves the inositol 1,4,5-triphosphate receptor (IP3R), glucose- regulated protein 75 (GRP75), and voltage-dependent anion channel (VDAC) complex, which participates in the transfer of Ca^2+^ from the ER to the mitochondria [24]. Silencing Nogo-B receptors impaired mitochondrial dysfunction, including reduced Ca^2+^ uptake, respiration, and superoxide production, while increasing, activating the HIF-1α and promoting excessive VSMC proliferation [25]. Overexpression of the ER-phagy receptor FAM134B mediates Ca^2+^ transfer from ER to mitochondria, increasing apoptosis, impairing invasion, and causing mitochondrial dysfunction in PE [26]. Our functional assays revealed that Calpastatin overexpression promotes trophoblast proliferation, migration, and invasion under normoxia and hypoxia. Knockdown significantly impaired these functions under normoxia. Under hypoxia, proliferation differences were non-significant (likely due to inherently slower proliferation), but migration clearly decreased at 48 h, and invasion decreased at both time points. Ca^2+^ analysis showed overexpression reduced intracellular Ca^2+^, while knockdown increased it. Thus, calpastatin reduction may lead to calcium overload which can activate calcium-dependent proteases (e.g., calpain), inducing mitochondrial permeability transition pore (mPTP) opening, consequently inhibiting cell migration and promoting apoptosis. Thus, calpastatin likely maintains Ca^2+^ homeostasis by regulating ER-mitochondrial calcium cycling via the IP3R-GRP75-VDAC pathway, thereby reducing apoptosis and enhancing trophoblast cell motility. Thus, calpastatin reduction may increase Ca^2+^ content in the mitochondria-associated membrane, leading to trophoblast cell dysfunction. Hypoxia also induces mitochondrial fission, possibly by activating Drp1 through HIF-1α [27]. PE exhibit disrupted mitochondrial dynamics with increased fission and reduced OPA1 expression [28]. OPA1, crucial for inner membrane fusion, promotes endothelial mitochondrial fusion and angiogenesis [29]. In this experiment, knockdown significantly upregulates fusion protein Mfn2. mirroring PE the placental pathology [30]. The resulting mitochondrial fragmentation directly impairs trophoblast proliferation by 35–40% and migration by approximately 50%.Targeting mitochondrial dynamics may ameliorate PE [31].Autophagy is crucial for maintaining cellular homeostasis.Autophagy imbalance contributes to PE pathogenesis by affect vascular remodeling [32]. Studies have shown that autophagic regulators, such as PKCβ, are reduced in PE placentas, and PKCβ inhibition induces PE-like symptoms through autophagy activation. The upregulation of sFlt-1 and downregulation of VEGF-A in mouse placentas, as well as the imbalance between pro-angiogenic and anti-angiogenic factors, leads to PE development [33]. Autophagy also regulates Treg differentiation, influencing immune responses in PE [34]. Several autophagy-related proteins participate in autophagy. PINK1-mediated is reduced in PE placentas. PINK1 overexpression alleviates hypoxia-induced ROS and necroptosis [35]. In vitro PE models show elevated autophagy markers (P62,LC3), and inhibitors suppress cell invasion/migration [36], suggesting increased autophagy plays an important role in the pathogenesis of PE. In our experiments, In our HTR-8/SVneo cells under hypoxia, autophagy proteins PINK1/Parkin decreased. Overexpression increased their expression, suggesting a potential autophagy deficiency in PE. Overexpression reduced apoptosis, while knockdown increased it. JC-1 staining confirmed reduced mitochondrial membrane potential and ATP in the knockdown cells, initially maintain autophagic dominance via autophagy, reducing apoptosis. Later, autophagy promotes apoptosis via pathways such as p53 and BH3 [37]. Mitophagy prevents apoptosis by reducing mitochondrial outer membrane permeabilization (MOMP) and pro-apoptotic proteins release such as cytochrome C and SMAC/DIABLO, creating a counterbalance between autophagy and apoptosis [38].The observed decrease in autophagy and increase in apoptosis in vitro may reflect an inability to sustain autophagy under hypoxic stress, leading to apoptosis that further inhibits autophagy. Calpastatin knockdown-induced ATP depletion likely compromise cellular energy metabolism, impairing migration and invasion. Concurrently, the diminished MMP could trigger cytochrome C release, subsequently activating caspase cascade pathways and promoting apoptosis. Conversely, calpastatin overexpression enhances MMP, which not only facilitates ATP production but also strengthens anti-apoptotic mechanisms, ultimately reducing apoptosis. These alterations align with the characteristic mitochondrial dysfunction, ER stress, and increased apoptosis observed in PE [5, 39], suggesting synchronized mitochondrial-ER dysregulation contributes to PE pathogenesis Therefore, inhibiting apoptosis to maintain mitochondrial-ER function may be a target for the treatment.

Notably, our in vitro model lacks validation in vivo validation experiments. But the H/R model reveals cell-autonomous hypoxia responses distinct from systemic PE pathology, these findings have important implications. Future work should involve animal models to explore Calpastatin's impact on placental development, blood pressure, and PE symptoms, comprehensively evaluating its therapeutic potential and safety. Calpastatin could also serve as a biomarker for PE risk prediction, which is of great significance for predicting, controlling, and delaying the onset of PE.

## Conclusion

This study successfully established a hypoxia/reoxygenation model in HTR8-Svneo trophoblasts, identifying 12 h hypoxia as critical point. We thoroughly investigated the role of calpastatin in regulating trophoblast function and mitochondria-ER homeostasis under hypoxic. Results demonstrate that calpastatin significantly promotes trophoblast proliferation, invasion, migration, and inhibits apoptosis. Knockdown specifically impairs mitochondria-ER functional integrity by increasing the Mfn2, decreasing PINK1, elevating GRP78, resulting ATP production, causing Ca^2+^ overload, and inducing of mitochondrial membrane potential loss These findings strongly suggest calpastatin is a key protective factor enabling trophoblasts to adapt to hypoxic. Its downregulation or impairment likely contribute to trophoblast dysfunction in PE pathogenesis by disrupting mitochondria-ER homeostasis.

## Data Availability

All data generated or analysed during this study are included in this published article.
